# Supplementary material for: Molecular basis of collagen triple helix recognition by VWF A-like domain two of collagen VII: Implications for interlaced anchoring fibril formation
Source: J Biol Chem. 2026 Aug 13;302(10):113448. doi: 10.1016/j.jbc.2026.113448 (PMC13599636; doi:10.1016/j.jbc.2026.113448)
Supplement: Supplementary file 1 [file mmc1.docx]

**Supporting Information**

**Molecular basis of collagen triple helix recognition by VWF A-like domain 2 of collagen VII: Implications for** **interlaced anchoring fibril formation**

Moe Hashimoto, Hiroya Oki, Kazuki Kawahara, Kazunori K. Fujii, Takaki Koide

**Table of contents**

1. The two-hybrid interaction of the AD-fusion peptides with the triple-helical random peptide library and the BD-fusion collagen VII A2 domain, HSP47, and PEDF. (Figure S1)	S-2
2. Amino acid sequences of synthesized peptides. (Table S1)	S-3–S-4
3. Reverse-phase high-performance liquid chromatography (RP-HPLC) profiles of synthesized peptides. (Figure S2)	S-5–S-6
4. Mass spectrometry (MS) charts of the synthetic peptides. (Figure S3)	S-7–S-15
5. Circular dichroism (CD) profiles of peptides at 4°C (solid line), 25°C (dashed line), and 85°C (dash-dotted line). (Figure S4)	S-16–S-17
6. CD spectra recorded at 225 nm from 4 to 85°C. (Figure S5)	S-18
7. Predicted melting temperatures (*T*_m_) of triple-helical peptides containing Trp at Xaa3. (Table S2)	S-18
8. Collagen VII A2 binding increased with the proportion of the C3-Pep 4 in the peptide polymer. (Figure S6)	S-19
9. Data collection and refinement statistics. (Table S3)	S-20
10. Co-crystal of collagen VII A2 and Short-XGWXGA. (Figure S7)	S-21
11. Collagen VII A2 domain recognizes Nle-containing triple-helical peptide. (Figure S8)	S-22
12. Structural comparison of collagen VII A2 between the apo and complex forms. (Figure S9)	S-23
13. Collagen VII A2 recognizes the collagen *triple-helical* peptides in a metal ion-independent manner. (Figure S10)	S-24
14. Comparison of collagen-binding modes of collagen VII A2 and VWF A3. (Figure S11)	S-25
15. SDS-PAGE analysis of purified GST-collagen VII A2 domain. (Figure S12)	S-26
16. References	S-26–S-27

**Figure S1. The two-hybrid interaction of the AD-fusion peptides with the triple-helical random peptide library and the BD-fusion collagen VII A2 domain, HSP47, and PEDF.** The cells were cultured at 25°C for 4 days. The interaction allows yeast cells to survive and turn blue by degrading X-α-Gal. Seq *a* is Lys-Gly-His-Arg-Gly-Phe-Ser-Gly-Leu (1).

**Table S1. Amino acid sequences of synthesized peptides.**

| **Name** | **Sequence** |
| --- | --- |
| Pep 0  Pep 1  Pep 2  Pep 3  Pep 4  Pep 5  Pep 6  C3-Pep 4  C3-Pep 5  C3 | H-(Pro-Hyp-Gly)_6_-Pro-Arg-Gly-(Pro-Hyp-Gly)_5_-Pro-Tyr-NH_2_  H-(Pro-Hyp-Gly)_4_-Pro-Met-Gly-Phe-Gln-Gly-Leu-Arg-Gly-Asp-Hyp-Gly-(Pro-Hyp-Gly)_4_-Pro-Tyr-NH_2_  H-(Pro-Hyp-Gly)_4_-Pro-Met-Gly-Phe-Met-Gly-Ala-Met-Gly-Ala-Hyp-Gly-(Pro-Hyp-Gly)_4_-Pro-Tyr-NH_2_  H-(Pro-Hyp-Gly)_4_-Pro-Met-Gly-Trp-Met-Gly-Ala-Lys-Gly-Arg-Hyp-Gly-(Pro-Hyp-Gly)_4_-Pro-Tyr-NH_2_  H-(Pro-Hyp-Gly)_4_-Pro-Met-Gly-Phe-Hyp-Gly-Pro-Lys-Gly-Asn-Hyp-Gly-(Pro-Hyp-Gly)_4_-Pro-Tyr-NH2  H-(Pro-Hyp-Gly)_5_-Pro-Arg-Gly-Gln-Hyp-Gly-Val-Met-Gly-Phe-Hyp-Gly-(Pro-Hyp-Gly)_5_-Pro-NH_2_  H-(Pro-Hyp-Gly)_5_-Phe-Hyp-Gly-Met-Arg-Gly-(Pro-Hyp-Gly)_5_-Pro-Tyr-NH_2_ (ref. 2)  H-Cys-Cys-Cys-(Pro-Hyp-Gly)_5_-Pro-Met-Gly-Phe-Hyp-Gly-Pro-Lys-Gly-Asn-Pro-Gly-(Pro-Hyp-Gly)_5_-Cys-Cys-Cys-OH  H-Cys-Cys-Cys-(Pro-Hyp-Gly)_5_-Pro-Arg-Gly-Gln-Hyp-Gly-Val-Met-Gly-Phe-Hyp-Gly-(Pro-Hyp-Gly)_5_-Cys-Cys-Cys-OH  H-Cys-Cys-Cys-(Pro-Hyp-Gly)_5_-(Pro-Pro-Gly)_2_-Pro-Arg-Gly-Pro-Pro-Gly-(Pro-Hyp-Gly)_5_-Cys-Cys-Cys-OH (ref. 3) |

(^D^Pro indicates D-Pro.)

Table S1. (*Continued*).

| **Name** | **Sequence** |
| --- | --- |
| ss-Pep 4  Pep 3-X  Pep 3-1  Pep 3-2  Pep 3-3  Pep 3-4  Pep 3-5  Pep 3-6  Pep-XGWXGA  Short-XGWXGA | H-(Pro-Hyp-Gly)_2_-^D^Pro-Hyp-Gly-Pro-Hyp-Gly-Pro-Met-Gly-Phe-Hyp-Gly-Pro-Lys-Gly-Asn-Hyp-Gly-(Pro-Hyp-Gly)_2_-^D^Pro-Hyp-Gly-Pro-Hyp-Gly-Pro-Tyr-NH_2_  H-(Pro-Hyp-Gly)_4_-Pro-Nle-Gly-Trp-Nle-Gly-Ala-Lys-Gly-Arg-Hyp-Gly-(Pro-Hyp-Gly)_4_-Pro-Tyr-NH_2_  H-(Pro-Hyp-Gly)_4_-Pro-Hyp-Gly-Trp-Met-Gly-Ala-Lys-Gly-Arg-Hyp-Gly-(Pro-Hyp-Gly)_4_-Pro-Tyr-NH_2_  H-(Pro-Hyp-Gly)_4_-Pro-Met-Gly-Pro-Met-Gly-Ala-Lys-Gly-Arg-Hyp-Gly-(Pro-Hyp-Gly)_4_-Pro-Tyr-NH_2_  H-(Pro-Hyp-Gly)_4_-Pro-Met-Gly-Trp-Hyp-Gly-Ala-Lys-Gly-Arg-Hyp-Gly-(Pro-Hyp-Gly)_4_-Pro-Tyr-NH_2_  H-(Pro-Hyp-Gly)_4_-Pro-Met-Gly-Trp-Met-Gly-Pro-Lys-Gly-Arg-Hyp-Gly-(Pro-Hyp-Gly)_4_-Pro-Tyr-NH_2_  H-(Pro-Hyp-Gly)_4_-Pro-Met-Gly-Trp-Met-Gly-Ala-Hyp-Gly-Arg-Hyp-Gly-(Pro-Hyp-Gly)_4_-Pro-Tyr-NH_2_  H-(Pro-Hyp-Gly)_4_-Pro-Met-Gly-Trp-Met-Gly-Ala-Lys-Gly-Pro-Hyp-Gly-(Pro-Hyp-Gly)_4_-Pro-Tyr-NH_2_  H-(Pro-Hyp-Gly)_4_-Pro-Nle-Gly-Trp-Nle-Gly-Ala-Hyp-Gly-Pro-Hyp-Gly-(Pro-Hyp-Gly)_4_-Pro-Tyr-NH_2_  Ac-(Gly-Pro-Hyp)_2_-Gly-Pro-Nle-Gly-Trp-Nle-Gly-Ala-Hyp-(Gly-Pro-Hyp)_2_-NH_2_ |

**Figure S2. Reverse-phase high-performance liquid chromatography (RP-HPLC) profiles of synthesized peptides.** Column: Cosmosil 5C18-ARII (4.6 i.d. × 250 mm). Gradient: 10%–40% CH_3_CN in 0.05% TFA/H_2_O. over 30 min at 60°C. Flow rate: 1.0 mL/min. Absorbance: 220 nm.

Figure S2. (*Continued*).

**Pep 0**

calcd. MS [M_m_ + 3H]^3+^: 1176.227	found: 1176.228

**Pep 1**

calcd. MS [M_m_ + 3H]^3+^: 1215.005	found: 1215.237

**Figure. S3. Mass spectrometry (MS) charts of the synthetic peptides.**

**Pep 2**

calcd. MS [M_m_ + 3H]^3+^: 1179.197	found: 1179.198

**Pep 3**

calcd. MS [M_m_ + 3H]^3+^: 1219.573	found: 1219.569

Figure S3. (*Continued*).

**Pep 4**

calcd. MS [M_m_ + 3H]^3+^: 1195.225	found: 1195.223

**Pep 5**

calcd. MS [M_m_ + 4H]^4+^: 1000.475	found: 1000.476

Figure S3. (*Continued*).

**C3-Pep 4**

calcd. MS [M_m_ + 3H]^3+^: 1487.615	found: 1487.610

**C3-Pep 5**

calcd. MS [M_m_ + 4H]^4+^: 1130.972	found: 1130.974

Figure S3. (*Continued*).

**ss-Pep 4**

calcd. MS [M_m_ + 3H]^3+^: 1195.225	found: 1195.229

**Pep 3-X**

calcd. MS [M_m_ + 3H]^3+^: 1207.602	found: 1207.605

Figure S3. (*Continued*).

**Pep 3-1**

calcd. MS [M_m_ + 3H]^3+^: 1213.576	found: 1213.577

**Pep 3-2**

calcd. MS [M_m_ + 3H]^3+^: 1189.898	found: 1189.897

Figure S3. (*Continued*).

**Pep 3-3**

calcd. MS [M_m_ + 3H]^3+^: 1213.576	found: 1213.577

**Pep 3-4**

calcd. MS [M_m_ + 3H]^3+^: 1228.245	found: 1227.747

Figure S3. (*Continued*).

**Pep 3-5**

calcd. MS [M_m_ + 4H]^4+^: 911.170	found: 910.667

**Pep 3-6**

calcd. MS [M_m_ + 4H]^4+^: 900.170	found: 900.170

Figure S3. (*Continued*).

**Pep-XGWXGA**

calcd. MS [M_m_ + 3H]^3+^: 1182.904	found: 1182.906

**Short-XGWXGA**

calcd. MS [M_m_ + 2H]^2+^: 996.995	found: 997.005

Figure S3. (*Continued*).

**Figure S4. Circular dichroism (CD) profiles of peptides at 4°C (solid line), 25°C (dashed line), and 85°C (dash-dotted line).** A positive maximum at 225 nm indicates a polyproline II-like helical structure (4).

Figure S4. (*Continued*).

**Figure S5. CD spectra recorded at 225 nm from 4 to 85°C.** A cooperative decrease in the signal with increasing temperature indicates triple-helix melting, whereas a linear decrease reflects a single-chain polyproline II helix.

**Table S2. Predicted melting temperatures (*T*_m_) of triple-helical peptides containing Trp at Xaa3.**

| **Name** | **Sequence** | ***T*_m_ (°C) *^a^*** |
| --- | --- | --- |
| Clone 2    Clone 7    Clone 8 | H-(Pro-Hyp-Gly)_4_-Pro-Met-Gly-Trp-Trp-Gly-Pro-Ser-Gly-Thr-Hyp-Gly-(Pro-Hyp-Gly)_4_-Pro-Tyr-NH_2_  H-(Pro-Hyp-Gly)_4_-Pro-Met-Gly-Trp-Met-Gly-Ala-Lys-Gly-Arg-Hyp-Gly-(Pro-Hyp-Gly)_4_-Pro-Tyr-NH_2_  H-(Pro-Hyp-Gly)_4_-Pro-Met-Gly-Trp-Trp-Gly-Ala-Thr-Gly-Thr-Hyp-Gly-(Pro-Hyp-Gly)_4_-Pro-Tyr-NH_2_ | 9.7  32.9  17.2 |

*^a^* *T*_m_ value was predicted SCEPTTr (5).

**Figure S6. Collagen VII A2 binding increased with the proportion of the C3-Pep 4 in the peptide polymer.** GST-collagen VII A2 was incubated with ELISA wells coated with peptide polymers (0.5 μg/well) at 25°C. Percentages of polymers represent ratios of the C3-Pep 4 weight to the total peptide weight (C3-Pep 4 and C3). Bound protein was detected using HRP-conjugated anti-GST antibody and ABTS substrate, and absorbance was measured at 405 nm (n = 3, mean ± S.D.).

**Table S3. Data collection and refinement statistics.**

|  | Human collagen VII A2 domain–Short-XGWXGA peptide complex |
| --- | --- |
| **Data collection**  Beamline  Detector  Wavelength (Å)  Space group  Unit-cell parameters  *a, b, c* (Å)  α, β, γ (º)  Unique reflections  Resolution (Å)  *R_meas_* (%)  CC_1/2_ (%)  Completeness (%)  Average *I/σ* (I)  Redundancy  **Refinement**  Resolution (Å)  Reflections  No. Atoms  Protein  Waters  SO4  *R_work_*  *R_free_*  R.M.S.D. from ideal  Bonds (Å)  Angles (º)  *B*-factors (Å^2^)  Ramachandran plot analysis (%)  Most favored  Allowed | SPring-8 BL45XU  EIGER X 16M  1.0  P2_1_  72.01, 37.17, 80.49,  90.00, 115.77, 90.00  65,405 (3,152)  40.39-1.47 (1.50-1.47)  30.6 (124.5)  99.3 (60.0)  99.3 (97.3)  15.6 (2.3)  6.8 (7.0)  40.39-1.47 (1.49-1.47)  65,389 (2,093)  3,745  606  10 (2 molecules)  0.176 (0.316)  0.217 (0.370)  0.008  1.034  19.00  96.17  3.83 |

**Figure S7. Co-crystal of collagen VII A2 and Short-XGWXGA.** Purified A2 domain (243 μM) and Short-XGWXGA peptide (291 μM) were mixed, and crystallization screening was performed at 4°C. Diffraction-quality plate-like crystals were obtained in reservoir solution containing 26% (w/v) PEG 4000, 100 mM sodium acetate, and 100 mM ammonium sulfate at pH 4.8.

**Figure S8. Collagen VII A2 domain recognizes Nle-containing triple-helical peptide.** *A*, Binding of 10 nM GST-collagen VII A2 to wells coated with 100% C3-Pep 4 polymer (0.5 μg/well) was inhibited by Pep 3 (solid line) or Pep 3-X (dashed line). The experiment was conducted at 25°C (n = 3, mean ± S.D.). *B*, Binding of 10 nM GST-collagen VII A2 to wells coated with 100% C3-Pep 4 polymer (0.5 μg/well) was inhibited by Pep 3 (solid line) or Pep-XGWXGA (dashed line). The experiment was conducted at 25°C (n = 3, mean ± S.D.).

**Figure S9. Structural comparison of collagen VII A2 between the apo and complex forms.** Collagen VII A2 is shown as a cartoon model, and Short-XGWXGA as a stick model. The leading, middle, and trailing chains of Short-XGWXGA are colored cyan, yellow, and magenta, respectively. The A2 apo form (PDB: 6S4C) (6) is shown in orange, and the A2 complex form is shown in green. The RMSD is 0.506 Å.

**Figure S10. Collagen VII A2 recognizes the collagen *triple-helical* peptides in a metal ion-independent manner.** *A*, GST-collagen VII A2 was incubated with ELISA wells coated with 100% C3-Pep 4 polymer (0.5 μg/well) at 25°C in the presence of MgCl₂ (solid line) or EDTA (dashed line). Bound protein was detected using HRP-conjugated anti-GST antibody and ABTS substrate. Absorbance was measured at 405 nm (n = 3, mean ± S.D.). *B*, Binding of 5 nM GST-collagen VII A2 to wells coated with 100% C3-Pep 4 polymer (0.5 μg/well) was inhibited by triple-helical Pep 4 (solid line) or single-stranded ss-Pep 4 (dashed line). Pep 4 that assumes triple-helical conformation at the assay temperature of 25°C, a stereoisomeric peptide containing D-Pro in place of L-Pro (ss-Pep 4), which fails to adopt a triple-helical conformation, was included as a competitor (Table S1). Circular dichroism analysis confirmed that ss-Pep 4 fails to form a triple-helical structure (Figure S4, Figure S5). Triple-helical Pep 0 served as a control. The experiment was conducted at 25°C (n = 3, mean ± S.D.).

**Figure S11. Comparison of collagen-binding modes of collagen VII A2 and VWF A3.** The structure of VWF A3 (red) in complex with the triple-helical peptide was taken from a reported model (PDB: 4DMU) (7). The structure of collagen VII A2 (green) corresponds to the A2 domain extracted from the co-crystal structure determined in this study.

**Figure S12. SDS-PAGE analysis of purified GST-collagen VII A2 domain.** Proteins were separated on a 12% acrylamide gel, and bands were visualized by CBB staining. The molecular size of GST-collagen VII A2 domain is 48 kDa.

**SI References**

1. Masuda, R., Thant, K. P. P., Kawahara, K., Oki, H., Kadonosono, T., Kobayashi, Y., and Koide, T. (2024) A yeast two-hybrid system to obtain triple-helical ligands from combinatorial random peptide libraries. *J. Biol. Chem*. **300**, 107794
2. [preprint] Shibata, S. S., Oki, H., Kawahara, K., Masuda, R., Fujii, K. K., and Koide, T. (2025) Triple-helical ligands selectively targeting the closed αI domain of integrin α2β1. *bioRxiv*. 10.1101/2025.07.28.667101
3. Ichise, S. F., Takeushi, S., Aoki, S., Kuroda, K. C., Nose, H., Masuda, R., and Koide, T. (2019) Development of a collagen-like peptide polymer *via* end-to-end disulfide cross-linking and its application as a biomaterial. *Acta Biomater*. **94**, 361-371
4. Lopes, J. L. S., Miles, A. J., Whitmore, L., and Wallace, B. A. (2014) Distinct circular dichroism spectroscopic signatures of polyproline II and unordered secondary structures: Applications in secondary structure analyses. *Protein Sci*. **23**, 1765-1772
5. Walker, D. R., Hulgan, S. A. H., Peterson, C. M., Li, I. C., Gonzalez, K. J., and Hartgerink, J. D. (2021) Predicting the stability of homotrimeric and heterotrimeric collagen helices. *Nat. Chem*. **13**, 260-269
6. [dataset] Gebauer, J. M., Flachsenberg, F. Windler, C., Richer, B., Baumann, U., Seeger, K. (2019) Crystal Structure of the vWFA2 subdomain of type VII collagen. Protein Data Bank. 6S4C.
7. [dataset] Brondijk, T. H. C., Bihan, D., Farndale, R. W., and Huizinga, E. G. (2012) Crystal structure of the von Willebrand factor A3 domain in complex with a collagen III derived triple-helical peptide. Protein Data Bank. 4DMU.
